# SEISMIC-HF 1: key findings from AHA24 and implications for remote cardiac monitoring

**DOI:** 10.1007/s10741-025-10514-1

**Published:** 2025-04-23

**Authors:** Baljash Cheema, Anjan Tibrewala

**Affiliations:** 1 Center for Artificial Intelligence, Bluhm Cardiovascular Institute, Northwestern Medicine, 676 N. Saint Clair Street Arkes Pavilion, Suite 600, Chicago, IL 60611 USA; 2 Feinberg School of Medicine, Northwestern University, 676 N. Saint Clair Street Arkes Pavilion, Suite 600, Chicago, IL 60611 USA

**Keywords:** Heart failure, Wearable sensors, Machine learning, Artificial intelligence, Right heart catheterization

## Abstract

While there is continued progress in developing therapies for patients with heart failure, the condition results in significant morbidity and a sizeable economic impact on our society. Recent advances in wearable sensors combined with machine learning algorithms give hope that heart failure can be better managed remotely and allow for improved clinical outcomes. This is a focused review of the key findings of the SEISMocardiogram In Cardiovascular Monitoring for Heart Failure I (SEISMIC-HF 1) study, presented at the American Heart Association’s Scientific Sessions 2024 in Chicago, Illinois. This study showcased the ability of a machine learning algorithm to estimate pulmonary capillary wedge pressure in patients with heart failure with reduced ejection fraction, utilizing seismocardiography, photoplethysmography, and electrocardiography signals obtained non-invasively through a wearable sensor patch (CardioTag) for model input. The authors showed a significant correlation between model-predicted pulmonary capillary wedge pressure and the gold standard pressure measurement obtained from right heart catheterization. Future investigations should assess the implementation of this technology as a part of a treatment strategy for outpatient heart failure care and explore its performance in additional study populations including those with heart failure with preserved ejection fraction and in patients outside of the clinical environment.

## Introduction

Heart failure (HF) continues to be a major public health concern, with a high rate of morbidity and mortality for those suffering from the condition and a large incidence of hospitalizations or unscheduled visits to medical centers for HF treatment [1]. HF exacerbations often progress from an asymptomatic state with rising intracardiac filling pressures over the course of several weeks to the development of frank symptoms prompting acute medical care for management [2, 3]. This window of subclinical congestion provides a time period for intervention, where decongestive therapy may prevent the development of symptoms and the need for hospitalization, as seen in trials of HF management guided by pulmonary artery measurements from implantable sensors [4, 5]. Furthermore, there is data to suggest that hemodynamic-guided management with a pulmonary artery sensor may reduce mortality in patients with heart failure with reduced ejection fraction (HFrEF) [6]. Despite their clinical utility, use of these invasive devices has been limited by patient reluctance, lack of insurance coverage, and concern for complications.

Recent advances in sensor technology including miniaturization of device components, enhanced connectivity and communication, improved battery efficiency and storage, and integration with high-powered machine learning algorithms has led to optimism that similar levels of insight currently obtained through implantable devices can be created from non-invasive wearable sensors [7, 8]. As such, investigators recently presented early results from the SEISMocardiogram In Cardiovascular Monitoring for Heart Failure I (SEISMIC-HF I) study at the American Heart Association’s Scientific Sessions 2024 as Late-Breaking Science in Chicago, Illinois, showing that wearable sensor technology alongside machine learning can be combined to accurately estimate intracardiac filling pressures, in this case the pulmonary capillary wedge pressure (PCWP) for patients with HFrEF [9, 10].

## Study design

SEISMIC-HF 1 is a prospective, multi-site, observational study of 943 patients at 15 sites in the United States undergoing a standard of care, clinically indicated right heart catheterization (RHC) while simultaneously wearing a wearable sensor to obtain non-invasive signals for processing. Patients over the age of 21 with a history of HF or suspected HF who were already scheduled to undergo a RHC were eligible for the study. Key exclusion criteria were patients receiving temporary or durable mechanical circulatory support, on mechanical ventilation, with allergies to components of the wearable sensor, with open chest wounds that may interfere with device readings or pose risks associated with wound healing, or who were hemodynamically unstable and not suitable for study enrollment.

**Fig. 1:**
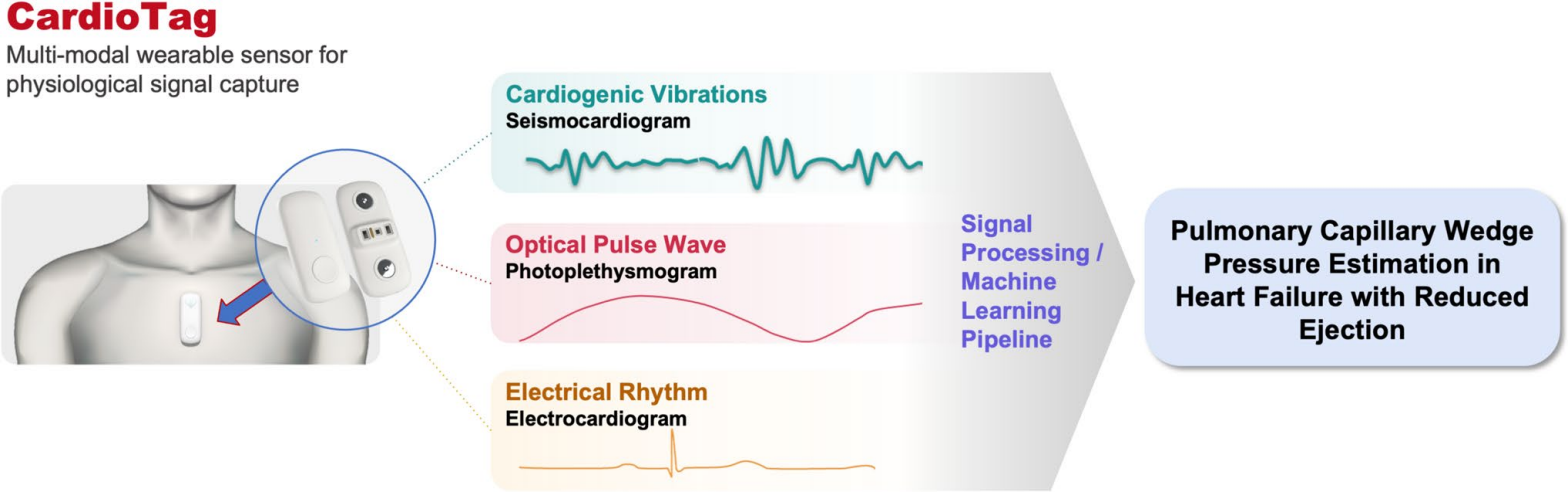
Physiologic signals from non-invasive sensor for pulmonary capillary wedge pressure estimation. CardioTag (Cardiosense, Inc., Chicago, IL) is a wearable sensor patch capable of capturing seismocardiography, photoplethysmography, and electrocardiography signals non-invasively. When combined with machine learning, this technology is able to non-invasively estimate pulmonary capillary wedge pressure in those with heart failure with reduced ejection fraction

The wearable sensor used was the CardioTag (Cardiosense, Inc., Chicago, IL), a medical-grade, multi-sensor device that can simultaneously capture seismocardiography, photoplethysmography, and electrocardiography signals (Fig. 1). Seismocardiography is a non-invasive method for measuring cardiac mechanical activity by detecting cardiac vibrations throughout the cardiac cycle. The idea that mechanical vibrations of the heart could be used as a surrogate for cardiac structure and function has been in existence since the 1950 s, but there has been a recent resurgence in interest in seismocardiography when these mechanical vibrations could be picked up and measured accurately by small, lightweight wearable sensors [11]. Photoplethysmography is a non-invasive method of measuring changes in blood volume and flow in the microvasculature, and electrocardiography measures the voltage over time due to myocardial electrical activity, collectively allowing the device to time components of the cardiac cycle.

In this specific analysis, the investigators limited the study cohort to those with HFrEF defined by ejection fraction ≤ 40%, which was 310 of the total 943 patients. The model focused on HFrEF given unique features of model development in HFrEF versus heart failure with preserved ejection fraction (HFpEF) and feedback from the Food and Drug Administration. After excluding studies that did not pass quality control, the remaining 233 were split 80/20 into a training set and a holdout set to train and measure the performance of a machine learning algorithm to estimate PCWP, using the right heart catheterization as the gold standard.

## Results

The patient population studied had a mean age of 61.1 ± 13.4 years, 39% were Black, and 38% were female. 57% had New York Heart Association Class III symptoms, while 16% had New York Heart Association Class IV symptoms. There was a high prevalence of hypertension, obesity, prior myocardial infarction or coronary artery disease, and chronic kidney disease. The mean PCWP on RHC was 18.1 ± 9.4 mmHg.

The investigators found a correlation between their model predicted PCWP and gold standard measurement on RHC in the holdout set (*r* = 0.74). This yields an *R*^2^ of 0.55, suggesting about 55% of the variation in PCWP by RHC was detected from their non-invasive sensor combined with the machine learning algorithm. The mean difference was 1.04 ± 5.57 mmHg. The Limit of Agreement from the Bland–Altman plot analysis was 11.9 mmHg for the upper bound and − 9.9 mmHg for the lower bound. Their results were stable across various subpopulations of interest, although the sample size did not allow for rigorous statistical exploration.

### Study implications and future directions

Overall, the authors showed a moderate correlation between a non-invasive sensor derived estimate of PCWP in a clinical setting compared to the gold standard of PCWP measured via RHC. It should be noted that the degree of correlation and margin of error in this study is in line with the performance of implantable pulmonary artery sensors in predicting pulmonary artery diastolic pressure [4, 5].

This study represents an important step forward in the quest of using non-invasive hemodynamic-guided management of HF patients to improve cardiovascular outcomes. Given the ideal use of this device is in remote settings similar to the role of implantable pulmonary artery pressure sensors, performance of the technology needs to be assessed outside the confines of the hospital. Additionally, although this study was limited to those with HFrEF, HFpEF constitutes at least 50% of patients with HF with equally poor outcomes, thus showing that this technology works in that population could yield great benefits to patients and clinicians alike. Lastly, it will be important to show that this technology can be implemented with prescriptive guidance to potentially impact medical management of individual patients. Without this, the technology risks suffering the fate of many other artificial intelligence based technologies that work well in silico but fail to impact clinical care in a meaningful way [12].

## Data Availability

No datasets were generated or analysed during the current study.
